# Enhanced breakdown strength and suppressed dielectric loss of polymer nanocomposites with BaTiO_3_ fillers modified by fluoropolymer

**DOI:** 10.1039/c9ra10591c

**Published:** 2020-02-17

**Authors:** Jianxin Zhang, Jiachen Ma, Luqing Zhang, Chuanyong Zong, Anhou Xu, Yabin Zhang, Bing Geng, Shuxiang Zhang

**Affiliations:** Shandong Provincial Key Laboratory of Fluorine Chemistry and Chemical Materials, School of Chemistry and Chemical Engineering, Shandong Engineering Research Center for Fluorinated Material, University of Jinan Jinan 250022 China chm_zhangyb@ujn.edu.cn

## Abstract

The introduction of ceramic fillers into a polymer matrix is an effective way to obtain dielectric nanocomposites with high energy storage density. However, the inorganic fillers are difficult to disperse evenly into the polymer matrix because of the poor compatibility, which stems from the large surface energy difference and the mismatch in dielectric constant between the fillers and polymer matrix. Polymer nanocomposites with high dielectric constant while maintaining high breakdown strength have great potential to achieve high energy storage density. In this work, poly(dodecafluoroheptyl methacrylate) terminated with a thiol end group (PDFMA-SH) was synthesized *via* a two-step process including Reversible Addition-Fragmentation Chain Transfer (RAFT) polymerization and subsequent aminolysis reaction. The polymer was then grafted into the surface of BaTiO_3_ (BT) nanoparticles by a “thiol–ene” click reaction to reduce the surface energy of BT nanoparticles. A novel nanocomposite consisted of the core–shell structured PDFMA@BT hybrid nanoparticles and poly(vinylidene fluoride–chlorotrifluoroethylene) (P(VDF–CTFE)) matrix was prepared. The influence of the fluoropolymer shell on the dispersion of fillers, the compatibility between the fillers and polymer matrix, dielectric properties and breakdown strength were investigated systematically. The results indicate that the strong interfacial adhesion between the hybrid nanoparticles and P(VDF–CTFE) matrix makes the fillers uniformly dispersed in the polymer matrix. Meanwhile, the excellent compatibility between the two components is favorable for enhancing the breakdown strength and suppressing dielectric loss, providing a condition to prepare dielectric materials with high energy storage density.

## Abstract 2

Preparation of high-performance dielectric composite films using PDFMA@BT hybrid nanoparticles as fillers.

## Graphical abstract

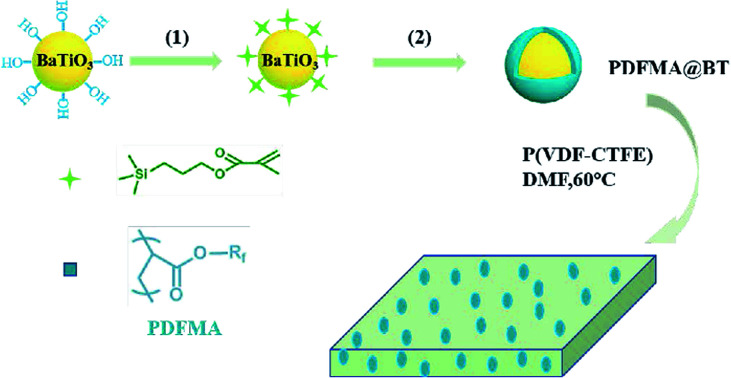

## 1. Introduction

Dielectric materials with high energy storage density have received great attention due to their wide application in capacitors, pulse power devices, hybrid electric vehicles, and vibration energy collectors.^1–6^ The energy storage density of the dielectrics is determined by the dielectric constant^7^ (*κ*) and breakdown strength (*E*_b_) of the nanocomposites. The energy storage density (*U*_e_) for linear dielectrics can be calculated by *U*_e_ = 1/2*ε*_r_*ε*_0_*E*_b_^2^, where *ε*_r_ and *ε*_0_ are the relative permittivity of the dielectrics and the vacuum permittivity,^8^ respectively. Therefore, *U*_e_ is determined by the synergy of the breakdown strength and dielectric constant, implying the high energy storage density can be achieved while the dielectric constant and breakdown strength are enhanced simultaneously.

In order to simultaneously increase the *E*_b_ and the dielectric constant, it is proposed to prepare a multi-component dielectric nanocomposite. One approach is to incorporate a conductive filler^9–12^ (such as aluminum powder, graphene, carbon nanotubes, *etc.*) into the polymer matrix. When the fillers concentration approaches the percolation threshold,^13^ an ultra-high dielectric constant can be achieved. However, the high dielectric constant is often accompanied by high dielectric loss and low breakdown strength, limited the achievable high energy storage density.^14,15^ Another effective method is to mix a high dielectric constant ceramic material^16–20^(such as BaTiO_3_ (BT), Pb(Zr,Ti)O_3_ (PZT), TiO_2_) with polymer matrix. The dielectric properties of the polymer nanocomposites can be improved by adjusting the fillers contents. However, the nano-size inorganic fillers with large specific surface area and high surface energy tend to agglomerating, which limits the increase of energy density of the nanocomposite.^8,18^ Thus, the preparation of the polymer nanocomposites with outstanding dispersity and stability remains is urgently required.

Ferroelectric polymers^21–25^ poly(vinylidene fluoride) (PVDF) and its binary/terpolymers are often used as a polymer matrix for the preparation of high performance dielectric nanocomposite materials due to their high dielectric constant. Compared to hydrocarbon-based polymers, fluoropolymers have lower surface energy, resulting in less compatible with most organic and inorganic materials. Therefore, in order to solve the problem of the agglomeration of ceramic fillers and the interfacial compatibility between PVDF matrix and fillers, researchers have concentrated a lot of work on the surface modification of ceramic fillers. Song *et al.*^26^ have used the self-polymerization of dopamine to modify BT fibers, effectively improving the interfacial compatibility. Zhang *et al.*^8^ have prepared PVDF based nanocomposites with paraffin-coated BT nanoparticles. The nanocomposite possessed a high energy storage density about of 21.1 J cm^−3^ with 50 vol% fillers. These strategies of BT nanoparticle modification with alkane modifiers still have the issues of fillers aggregation in the polymer matrix. That's because the structural difference between the polymer shell layer of the fillers and the polymer matrix, creating obstacles to practical application to some extent. Achieving the excellent interfacial adhesion of the nanocomposites, associated with the interfacial coupling effect between the matrix and the modifier of fillers, is the key to improve both the dielectric properties and breakdown strength of the nanocomposites.^27,28^ It has been shown that a large difference in relative permittivity between an inorganic ceramic filler and the polymer matrix leads to an inhomogeneous electric field distribution, since electric fields tends to concentrate in phases of low permittivity.^29^ This can be achieved by introducing a low-permittivity polymer shell layer on the surface of the high-permittivity nanofiller to mitigate the permittivity mismatch between the filler particle and polymer matrix.^30^ Therefore, we designed and synthesized the core–shell structured hybrid fillers using fluoropolymer as the organic shells of fillers to reduce the surface energy of BT nanoparticles. The polymer shells can decrease the interface difference^31^ between the fillers and the matrix, which can effectively enhance the interfacial adhesion.

In this work, we firstly synthesized poly(dodecafluoroheptyl methacrylate) with a thiol end group (PDFMA-SH) by RAFT polymerization. Meanwhile, the double bonds functional groups were introduced to the BT surface by using a silane coupling agent. Subsequently, the PDFMA was grafted onto the surface of the BT nanoparticles by a “thiol–ene” click reaction to obtain PDFMA@BT hybrid fillers with a core–shell structure. The results show that the presence of fluoropolymer shells have improved the dispersibility of the fillers in the polymer matrix, and enhanced the compatibility between the fillers and the polymer matrix, which could significantly improve the breakdown strength of the nanocomposites. The novel hybrid fillers modified by fluoropolymer provide a strategy for realizing dielectric nanocomposite materials with high energy storage density.

## 2. Experimental

### 2.1 Materials

Poly (vinylidene fluoride-*co*-chlorotrifluoroethylene) (P(VDF–CTFE)) was purchased from Solvay Plastics and the molar fraction of CTFE units is 9%. BaTiO_3_ nanoparticles with an average diameter about of 100 nm were purchased from Shanghai Aladdin. Dodecafluoroheptylmethacrylate (DFMA) was purchased from Xeogia Fluorine-Silicone Chemical Co. Ltd (Harbin, China). Cumyldithiobenzoate (CDB) as a RAFT agent was purchased from Sigma-Aldrich. Methacryloxy propyl trimethoxyl silane (KH570) and 2,2-dimethoxy-2-phenylacetophenone (DMPA) are purchased from China Pharmaceutical Group Co. Ltd.

### 2.2 Synthesis of the thiol-terminated PDMFA

PDFMA–CDB^32^ is synthesized typically as follows. DFMA (5.0 g, 1.25 × 10^−2^ mol), a mixture of CDB (0.226 g, 8.3 × 10^−4^ mol), AIBN (0.028 g, 1.66 × 10^−4^ mol) and THF (5 mL) was charged sequentially in a single-necked flask, and remove the air from the bottle. The reactor was purged with nitrogen and then placed in a water bath at 65 °C. After 12 h, the product was purified by precipitating into excess methanol (three times), and suction filtration, then drying in a vacuum for 24 h to obtain a pink powder PDFMA–CDB (4.021 g, 80.7 wt% yield).

### 2.3 Modification of BT nanoparticles by fluoropolymer

PDFMA@BT with the core–shell structure was prepared as follows: 10.0 g BT nanoparticles were dispersed in 100 mL H_2_O_2_ (30 wt%) solution with ultrasonic treatment for 30 min, and then refluxed at 105 °C in an oil bath for 6 h. The BT nanoparticles were separated by centrifugation at 9000 rpm and washed with deionized water thoroughly, and then the product was dried at 60 °C for 24 h to obtain hydroxylated BT (BT-OH). Subsequently, 10.0 g of BT-OH nanoparticles were dispersed in 100 mL anhydrous toluene solution with ultrasonic treatment for 30 min. Then 2.0 g KH-570 and 1 mL acetic acid was added. The reaction system was heated to 80 °C for 12 h under the atmosphere of nitrogen. The nanoparticles were separated by centrifugation at 9000 rpm and washed with ethanol thoroughly, then the product was dried at 60 °C for 24 h to obtain vinyl-functionalized BT nanoparticles (BT-ene). Fluoropolymer-functionalized BT nanoparticles were obtained in the last step: 10.0 g of BT-ene nanoparticles and 100 mL of THF were added to a flask, and the mixture was sonicated for 30 min followed by the addition of PDFMA-SH (1.0 g) and DMPA (0.1 g). Under the protection of nitrogen, the dispersion was stirred at room temperature for 12 h with ultraviolet light irradiation (*λ* = 365 nm). After centrifugation and washed with THF, the nanoparticles were dried at 60 °C for 24 h to obtain PDFMA@BT.

### 2.4 Preparation of nanocomposite

The core–shell structured PDFMA@BT nanoparticles were firstly dispersed into DMF and sonicated for 30 min. P(VDF–CTFE) was then added with vigorous stirring, and the temperature was raised to 60 °C to form a stable suspension. The suspension was cast onto a glass substrate and the thickness of the film was controlled using a handy scraper. The cast film was dried under vacuum at 80 °C for 24 h to remove DMF. The resulting film has a thickness of about 10 μm.

## 3. Characterization

The ^1^H nuclear magnetic resonance (^1^H NMR) was characterized by an Advance III 400 MHz spectrometer at room temperature, and the deuterated acetone was used as a solvent. The molecular weight distribution (*Đ*) of the polymer was determined by gel permeation chromatography (GPC) with THF as the eluent (a flow rate of 1.0 mL min^−1^). UV-vis spectra were recorded on TU-1901 Spectrophotometer with a sampling interval of 1.00 nm. The analysis of the functional groups of BT nanoparticles was carried out by Fourier transform infrared spectroscopy (FTIR, Nicolet iS10). X-ray diffraction (XRD, PANalytical) was adopted to identify the phase composition of the PDFMA@BT nanocomposite. Transmission electron microscope (TEM) images of PDFMA@BT nanoparticles were obtained *via* a JEOL-1400 electron microscope. Scanning electron microscopy (SEM, S-2500, Hitachi Seiki) was used for the characterization of the dispersion of fillers in the nanocomposites. The dielectric properties were measured using a broadband dielectric spectrometer (Agilent E4990A) at the frequency range from 10^2^ Hz to 10^7^ Hz. The breakdown strength (*E*_b_) was characterized using a Programmable Withstanding Voltage Tester (CS9916AX) under a DC 500 V s^−1^ until sample failure.

## 4. Result and discussion

### 4.1 Preparation and characterization of thiol-terminated PDFMA

**Scheme 1:**
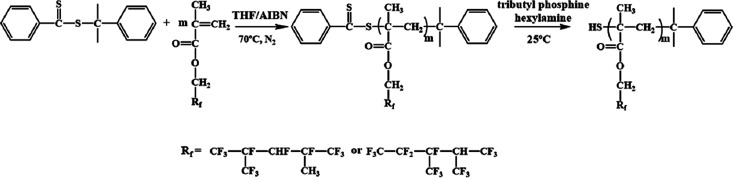
Preparation route of PDFMA-SH.

**Fig. 1:**
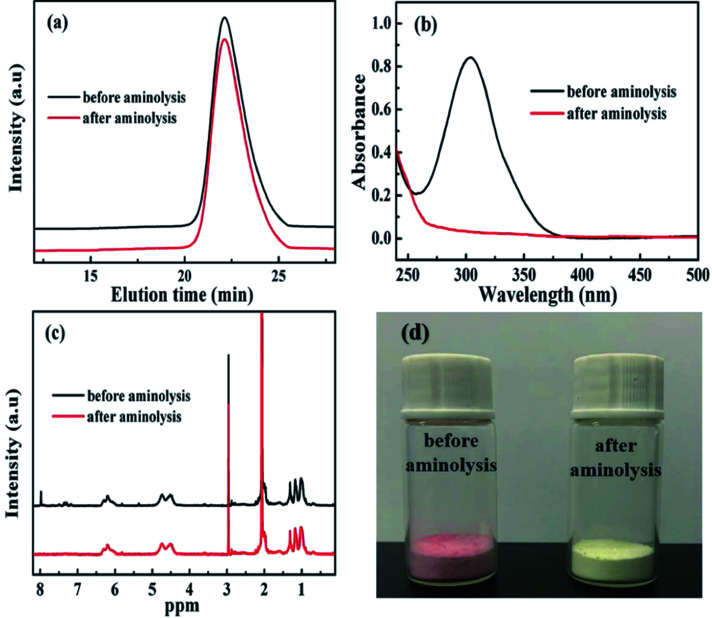
(a) GPC curves, (b) UV-vis spectra, (c) ^1^H NMR spectra, and (d) digital photograph of PDMFA before and after aminolysis.

The RAFT reagent CDB can effectively regulate the molecular weight and molecular weight distribution^32^ of PDFMA according to the previous reports. After polymerization, the polymer chains contain a thioester end group (Scheme 1), which can be converted into a thiol group by a reduction reaction. The thiol groups are used as an active site for grafting the polymer chain onto the surface of BT nanoparticles.^33^ As shown in Fig. 1a, the GPC curves of the homopolymers before and after the aminolysis are all single peaks, and the molecular weight distribution (∼1.1) is narrow. The GPC results certify that the disulfides are not formed during the aminolysis. Meanwhile, the ultraviolet absorption peak at 310 nm of the dithiocarbonate completely disappeared (Fig. 1b) after aminolysis, and the characteristic absorption of the phenyl (*δ* = 7.2–8.0 ppm) also disappeared (Fig. 1c). As a result, the color of the products is changed from pink to pale yellow as shown in Fig. 1d. According to the above results, the thioester groups have been successfully converted into thiol groups.

### 4.2 Preparation and characterization of PDFMA@BT nanoparticles

**Scheme 2:**
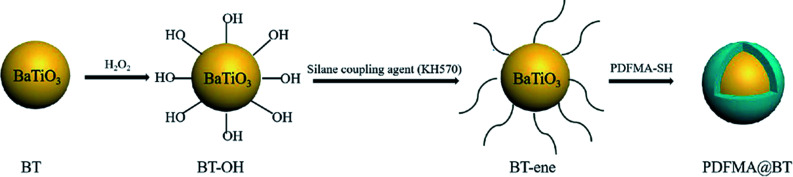
Preparation steps of PDFMA@BT nanoparticles.

**Fig. 2:**
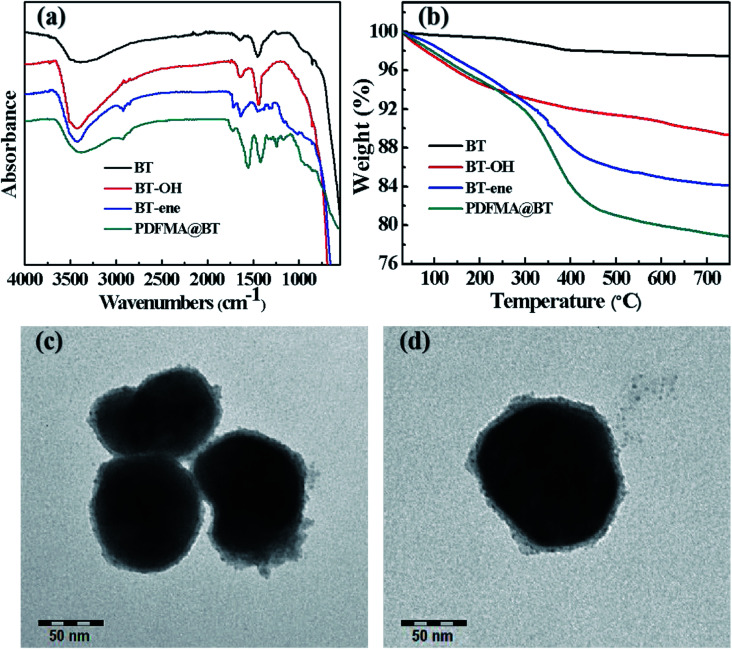
(a) FTIR spectra, (b) TGA curves of raw BT, BT-OH, BT-ene, PDFMA@BT and (c), (d) TEM images of PDFMA@BT nanoparticles.

Scheme 2 shows the preparation process of the core–shell structured PDFMA@BT hybrid nanoparticles. From the FTIR spectra as shown in Fig. 2a, a small amount of –OH groups (3400 cm^−1^) are presented in the raw BT, and the content of the –OH group is significantly increased after the hydroxylation with hydrogen peroxide. After that, the double bonds are introduced on the surface of BT-OH nanoparticles by the reaction of BT-OH with KH-570. New absorptions at 1040 cm^−1^, 1140 cm^−1^, 1720 cm^−1^ and 2800–3000 cm^−1^ corresponding to the stretching vibration of Si–O–Si, Si–O–Ba, C=O and –CH_2_– respectively appear compared with the raw BT nanoparticles, indicating that the silane coupling agent KH-570 is successfully grafted onto the surface of the BT nanoparticles. In order to obtain the core–shell structured nanoparticles PDFMA@BT, the “thiol–ene” click reaction between BT-ene and PMDFA-SH is initiated by the DMPA photoinitiator at room temperature under ultraviolet light (365 nm). The FTIR characterization is used to examine the functional groups of the obtained hybrid nanoparticles repeatedly washed by THF. The new absorption peaks at 1600 cm^−1^ and 1420 cm^−1^ originated from the vibration of the C–F bonds, confirming that the PDFMA was successfully grafted onto the surface of the BT nanoparticles.

Normally, the polymer will decompose at high temperature, but the BT nanoparticles will not show mass loss even above 1000 °C. Therefore, the TGA measurement can be used to analyze the amount of fluoropolymer grafted on the surface of BT nanoparticles. As shown in Fig. 2b, the weight loss of unmodified BT nanoparticles at 750 °C is only 2.55%, while the weight loss of BT-OH, BT-ene and PDFMA@BT gradually increase to 10.1%, 15.99%, and 21.5%, respectively. The weight loss of the modified BT nanoparticles under 300 °C may be caused by hydroxyl, solvent or other impurities in the sample, while the weight loss above 300 °C can be attributed to the decomposition of modifiers and fluoropolymers grafted onto the surface of the modified BT nanoparticles. Overall, it was confirmed by the TGA analysis that the content of the fluoropolymer grafted onto the surface of the BT nanoparticles was about 6 wt%.

The TEM images as shown in Fig. 2c and d further demonstrate the core–shell structured PDFMA@BT. It can be seen that the BT nanoparticles have an average size of about 100 nm. A rough polymer layer can be observed on the surface of the BT nanoparticles, and the thickness of the PDFMA shells was about ∼5 nm, which indicates that the core–shell structured PDFMA@BT nanoparticles are successfully synthesized.

### 4.3 Preparation and characterization of the nanocomposite

**Fig. 3:**
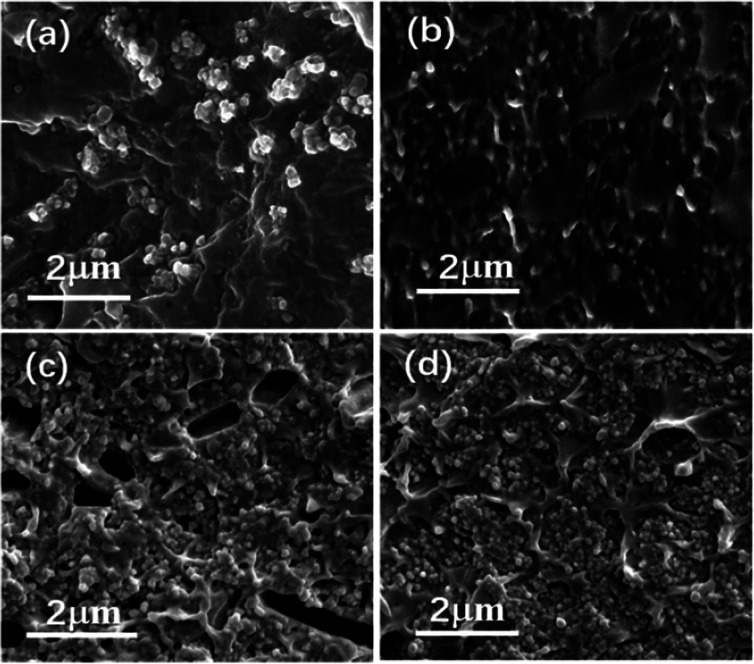
The cross-section SEM images of nanocomposite films with (a) 5 vol% BT nanoparticles, (b) 5 vol% PDFMA@BT nanoparticles, (c) 20 vol% BT nanoparticles and (d) 20 vol% PDFMA@BT nanoparticles.

Nanocomposites with different volume fraction (0–20 vol%) have been prepared to investigate the effects of the interfacial adhesion on the micromorphology and electrical properties. Fig. 3 shows the cross-sectional images of the two kinds of nanocomposites doping with different volume load with BT and PDFMA@BT fillers, respectively. It can be seen that the unmodified BT nanoparticles are poorly compatible with the P(VDF–CTFE) matrix as most fillers are exposed outside of the polymer matrix. In addition, the obvious agglomeration will induce the defects and disordered structures inside the films. In contrast, PDFMA@BT nanoparticles have a uniform dispersion in the P(VDF–CTFE) matrix without any agglomeration, which indicates that the excellent compatibility between PDFMA@BT and P(VDF–CTFE) matrix is constructed. The improvement of the compatibility can be attributed to the fact that PDFMA shells can reduce the surface energy of BT nanoparticles, increasing the distance between the nanoparticles. On the other hand, the strong interchain force between the fluoropolymer PDFMA and P(VDF–CTFE) matrix can undeniably enhance the interfacial adhesion between the fillers and polymer matrix.^27^

**Fig. 4:**
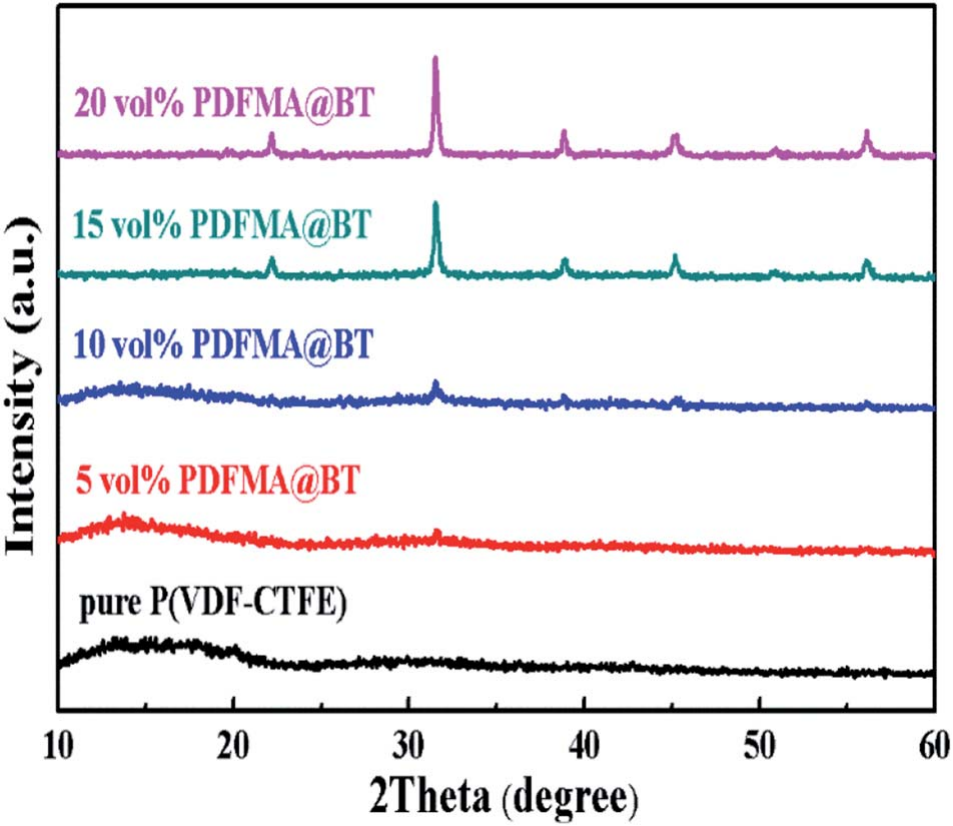
XRD patterns of PDFMA@BT/P(VDF–CTFE) nanocomposite films with different volume fractions.

Fig. 4 shows the XRD patterns of the PDFMA@BT/P(VDF–CTFE) nanocomposite films. The diffraction pattern of the pure P(VDF–CTFE) film exhibits a broad diffusion peak, and there is a small diffraction peak at 2*θ* = 20.2, indicating the typical amorphous polymer peaks. The diffraction at diffractogram of PDFMA@BT/P(VDF–CTFE) nanocomposite films is assigned to at 2*θ* values of about 22.22°, 31.63°, 38.88°, 45.34°, 50.63° and 56.25°, which assigned to the (100), (110), (111), (210), (211) and (220) diffractions of inorganic BT crystals, respectively. All the peaks are in accordance with ASTM data for perovskite BT (card 05-0626) and confirm the existence of inorganic BT nanoparticles in the polymer matrix. As the volume fraction of PDFMA@BT increases, the diffuse reflection of P(VDF–CTFE) in the nanocomposite film weakens and even disappears, and the intensity of the BT peak increases, which can be attributed to the compositional changes.^28^

**Fig. 5:**
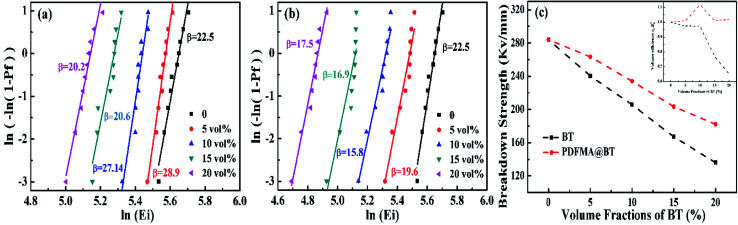
Weibull distribution curves of *E*_b_ of PDFMA@BT/P(VDF–CTFE) (a) and BT/P(VDF–CTFE) (b) nanocomposite films with various fillers loading. The evolutions of the *E*_b_ with volume contents of BT, PDFMA@BT at 1 kHz (c).

Breakdown strength is an important factor affecting the performance of dielectric materials. Fig. 5 shows the characteristic breakdown strength of the nanocomposite films, which is analyzed *via* two-parameter Weibull distribution function:

1 *P*(*E*) = 1 − exp[−(*E*/*E*_b_)^*β*^]

where *P*(*E*) is the cumulative probability of electrical faults. *β* is the shape parameter fitted by the distributed linear regression. *E* is the experimental breakdown strength, and *E*_b_ is the characteristic breakdown strength obtained when *P*(*E*) reaches 63.2%. This distribution function can convert into its logarithmic form:

2 log[−ln(1 − *P*)] = *β*(log *E* − log *E*_b_)

when the value of log[−ln(1 − P)] reaches zero, the *E* is equal to *E*_b_. Besides, for every specific value of *E*, *P* is calculated as follows:

3 *P* = (*i* − 0.44)/(*n* + 0.25)

where *i* indicates that this *E* value ranks the *i*th in the ascending order of breakdown strength data and *n* is the number of total data points.^34^

The *E*_b_ of the nanocomposite films with different fillers shows the same tendency as the volume fraction of the filler increases (Fig. 5a and b). As the volume fraction of BT nanoparticles increases, the fillers are more accessible to be agglomerated, causing the inevitable cracks and voids inside the nanocomposite films. The defects will sharply decrease the *E*_b_. The mismatch of the relative permittivity or conductivity between the inorganic filler and the polymer matrix usually leads to an uneven distribution of the electric field inside the composite, resulting in a significant reduction in the breakdown strength of the dielectric composite.^30^ It worth noting that PDFMA@BT/P(VDF–CTFE) nanocomposite films possess higher *β* value under the same volume fraction of fillers. It also proves the uniform dispersion of the fillers and the fewer defects inside the films from another aspect.

In order to study the effect of the fillers loading on the *E*_b_, the *E*_b_ at different volume fractions are calculated as shown in Fig. 5c. The *E*_b_ of the pure P(VDF–CTFE) matrix is 284.1 mV m^−1^, however, the *E*_b_ is drastically reduced to 136 mV m^−1^ for BT/P(VDF–CTFE) nanocomposite films when the fillers loading is 20 vol%. As for PDFMA@BT/P(VDF–CTFE) nanocomposite film, the PDFMA polymer shells greatly improve the compatibility of BT nanoparticles with P(VDF–CTFE) matrix and reduce the generation of leakage current channels, which are beneficial to increasing the *E*_b_. As shown in Fig. 5c, the curve of normalized energy density factor changing^35^ with the volume fraction of conductors also indicates that the PDFMA shell exhibits the ability to decrease the permittivity contrast between the BT filler and polymer matrix in the nanocomposite, and lessen the local electric field concentration, which improve the *E*_b_ of the PDFMA@BT/P(VDF–CTFE) nanocomposite film. As a result, the *E*_b_ of PDFMA@BT/P(VDF–CTFE) nanocomposite film with 20 vol% fillers loading achieves 188 mV m^−1^.

**Fig. 6:**
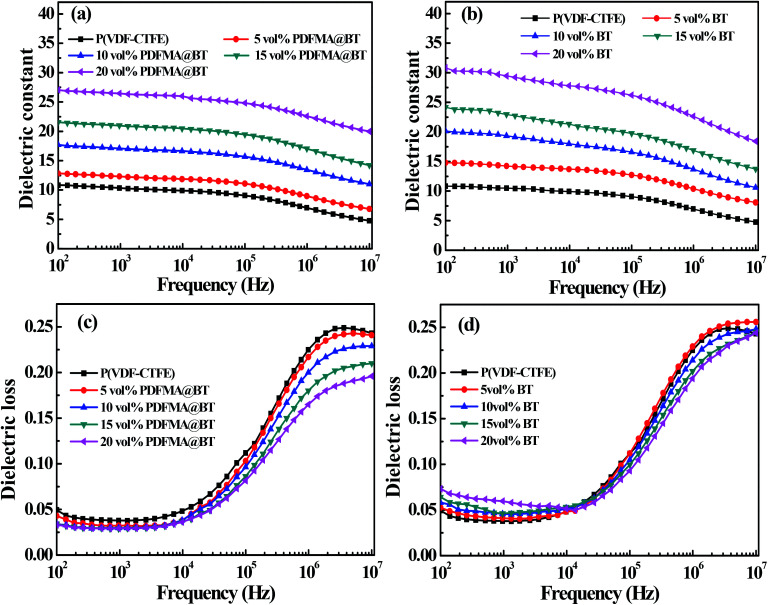
The frequency dependence of dielectric constant of PDFMA@BT (a) and BT (b) of nanocomposites with different fillers at room temperature. The frequency dependence of dielectric loss of PDFMA@BT (c) and BT (d) of nanocomposites with different fillers at room temperature.

Fig. 6 shows the dielectric properties of the different types of nanocomposite films with different fillers loading at room temperature. The dielectric constant of the nanocomposite films increases with the increase of fillers loading. For example, when the frequency is 100 Hz, the dielectric constant of PDFMA@BT/P(VDF–CTFE) nanocomposite film with 20 vol% fillers loading is 27.3, while the pure P(VDF–CTFE) film is 11. The reason is that BT nanoparticles have higher dielectric constant than that of the pure matrix. In addition, the increased content of the BT nanoparticles in the nanocomposite film can induce interfacial polarization and enhance the Maxwell–Wagner–Silars effect.^36,37^ As the frequency increases, the dielectric constant of the nanocomposite film gradually decreases and maintains relatively high stability at low frequency (10^2^ to 10^5^ Hz). In contrast, the dielectric constant of the nanocomposite film shows a significant decrease in the high-frequency range (10^5^ to 10^7^ Hz). It is because the speed of establishing interface polarization at high frequency cannot keep up with the change of the applied electric field, which leads to the sharp decrease of dielectric constant. Unfortunately, the PDFMA@BT/P(VDF–CTFE) nanocomposite film has a lower dielectric constant than that of BT/(PVDF-CTFFE) nanocomposite film under the same doping amount, especially in the low-frequency range. In addition, the PDFMA@BT/P(VDF–CTFE) nanocomposite film exhibits a weaker frequency dependency under the same condition compared with the BT/P(VDF–CTFE) nanocomposite film. Due to the weak polarization ability and low dependence on the frequency of the C–F bond in PDMFA, the modified BT nanoparticles can reduce the sensitivity to the electric fields due to the presence of the insulating PDMFA shells. Besides, the PDMFA shells can also enhance the interfacial adhesion between the fillers and matrix, which inhibits the interfacial polarization.

The polymer shells also show a positive effect on the dielectric loss of the nanocomposite. As shown in Fig. 6c and d, the dielectric loss of all nanocomposites are kept at a low level (<0.25). Compared with the pure P(VDF–CTFE) film, the dielectric loss of all nanocomposite films gradually decreases at the range of 10^4^ to 10^7^ Hz when the doping filler content increases. In the low-frequency range (10^2^ to 10^4^ Hz), although the dielectric loss of PDFMA@BT/P(VDF–CTFE) nanocomposite film has a slight increase than P(VDF–CTFE) film, there is a significant suppression compared with BT/P(VDF–CTFE) nanocomposites doping untreated BT. The inferior dielectric loss of BT/P(VDF–CTFE) nanocomposites is derived from the easy agglomeration of untreated BT nanoparticles in the polymer matrix and the poor compatibility between the two components, causing the stronger space-charge polarization and high dielectric loss.^17,31^ For the PDFMA@BT/P(VDF–CTFE) nanocomposite films, the modified BT nanoparticles with PDFMA shells can effectively improve the dispersibility of the BT nanoparticles in the P(VDF–CTFE) matrix and enhance the interfacial adhesion, which limits the macromolecular chain motion of the polymer matrix and the accumulation of charges.^38^

## 5. Conclusions

In summary, a facile “thiol–ene” click reaction between thiol groups functionalized PDFMA (PDFMA-SH) and olefinic bonds contained BT nanoparticles is used to reduce the surface energy of BT NPs and the corresponding PDFMA@BT/P(VDF–CTFE) nanocomposite films were prepared. The results show that the core–shell structured PDFMA@BT nanoparticles are more uniformly dispersed in the P(VDF–CTFE) matrix and exhibit good compatibility compared to the unmodified BT nanoparticles. When the content of nanoparticles is 20 vol%, the breakdown strength of PDFMA@BT/P(VDF–CTFE) nanocomposite film is 1.3 times that of BT/P(VDF–CTFE) nanocomposite film. In addition, PDFMA@BT/P(VDF–CTFE) nanocomposite films exhibit a significant suppression of dielectric loss. It is proved that the fluoropolymer shells have a positive effect on the improvement of the dielectric properties and breakdown strength.

## Conflicts of interest

Author declares no conflict of interest for this research paper.

## Supplementary Material
